# Influence of rs*1292037* Genetic Variant on miR‐21 Gene Expression in Patients With Type 1 Diabetes Mellitus: A Case‐Control Study

**DOI:** 10.1002/hsr2.70480

**Published:** 2025-03-02

**Authors:** Reza Bayat, Zivar Salehi, Setila Dalili, Farhad Mashayekhi

**Affiliations:** ^1^ Department of Biology, University Campus2 University of Guilan Rasht Iran; ^2^ Department of Biology, Faculty of Sciences University of Guilan Rasht Iran; ^3^ Pediatric Diseases Research Center Guilan University of Medical Sciences Rasht Iran

**Keywords:** microRNAs, miR‐21, rs1292037, SNP, type 1 diabetes mellitus

## Abstract

**Background and Aims:**

Alterations in the expression pattern of miRNAs seem to be linked with autoimmune diseases such as type 1 diabetes mellitus (T1DM). Regarding the importance of assessing this potential link, we aimed to evaluate the relationship between *miR‐21 rs1292037* single‐nucleotide polymorphism (SNP) and T1DM susceptibility. Furthermore, we investigated the *miR‐21* expression level in T1DM.

**Methods:**

A total of 250 T1DM patients and 250 controls were genotyped using polymerase chain reaction‐restriction fragment length polymorphism (PCR‐RFLP) and miR‐21 expression levels were assessed using real‐time PCR. Moreover, the potential targets of miR‐21 were investigated using different bioinformatics web servers.

**Results:**

Our results showed that the T/C genotype and the C allele were more frequent in T1DM patients than in controls. Individuals carrying the T/C genotype in overdominant model were 2.74‐fold at a higher risk of T1DM (OR = 2.74; 95%CI, 1.78–4.27; *p* < 0.0001). In addition, miR‐21 expression was more than twofold higher in patients than in controls (*p* < 0.0001) and it was found to be significantly upregulated when carrying the T/C genotype. Regarding miR‐21 predicted target genes, its overexpression may be associated with beta cell death, diabetic nephropathy, inflammatory responses, impaired insulin production or secretion, and T‐cell cytotoxicity, which are important in the initiation and progression of T1DM.

**Conclusion:**

Our results suggested that miR‐21 *rs1292037* may confer genetic susceptibility to T1DM. Therefore, it seems that this genetic link should be further investigated to enhance diagnostic and therapeutic strategies in these patients.

## 1 Introduction

Type 1 diabetes mellitus (T1DM) is a multifactorial autoimmune disease with a profound impact on individuals, their families, and society [1]. T1DM results from the selective loss of pancreatic β‐cells leading to insulin deficiency [2] and can severely affect multiple organs, particularly the heart, kidneys, and nerves [3]. It is well‐documented that genetic factors play a pivotal role in the incidence and pathogenicity of T1DM [4]. Epidemiological studies of T1DM have raised concerns due to its increasing prevalence worldwide [5]. It is estimated that 0.3% of children in the United States are diagnosed with T1DM annually [6]. It is well known that early diagnosis of T1DM can significantly prevent its complications and would increase the effectiveness of treatments, including stem cell transplantation and β‐cell regeneration strategies [7, 8].

MicroRNAs (miRNAs) are endogenous, evolutionarily conserved, single‐stranded molecules of approximately 22 nucleotides in length. They act in various biological processes by regulating gene expression through base‐pairing with various sites of their target mRNAs, particularly the 3' untranslated region (3'UTR) [9]. Studies have shown that miRNAs play a crucial role in the development and proper function of β‐cells [10].

Single nucleotide polymorphisms (SNPs) are the most common and fundamental form of genetic mutations that can contribute to the induction of devastating phenotypic outcomes [11]. It has been shown that SNPs in either the genes encoding miRNAs or in the 3'UTR of mRNAs can affect the binding affinity and ability of miRNAs and even alter the target mRNA [12]. Limited studies have shown the association between miRNA‐related SNPs with the incidence of several diseases, including T1DM [13].

To the best of our knowledge, a few studies have investigated the association between the miR‐21 rs1292037 SNP and human diseases [14, 15, 16, 17, 18, 19]. miR‐21 has emerged as a critical regulator of immune responses and inflammation, both of which are pivotal in the pathogenesis of T1DM. As highly conserved miRNAs, miR‐21 influences gene expression by targeting mRNAs involved in cell apoptosis, proliferation, and immune signaling [9, 13]. In T1DM, miR‐21 upregulation has been associated with the inhibition of T‐cell apoptosis, a process that contributes to the survival of autoreactive T‐cells, ultimately fostering autoimmunity and the destruction of pancreatic β‐cells [14, 20]. Moreover, miR‐21 enhances inflammatory responses through activation of pathways such as STAT3 and NF‐κB, both of which increase the expression of pro‐inflammatory cytokines, thereby intensifying β‐cell vulnerability to cytokine‐induced apoptosis [15, 20]. The role of miR‐21 in amplifying immune responses highlights its significance in T1DM development.

Minor allele frequency (MAF) is a critical parameter in SNP studies, representing the frequency of the less common allele in a given population. The MAF of rs1292037 has been reported at approximately 25%–30% in certain Middle Eastern and Asian populations, making it a suitable candidate for population‐based studies. MAF is crucial for understanding the potential impact of an SNP on disease susceptibility and for designing genetic association studies. A higher MAF increases the statistical power to detect associations with diseases, while population‐specific MAF variations can offer insights into genetic predispositions in different ethnic groups [21, 22].

The rs1292037 single‐nucleotide polymorphism (SNP) within the miR‐21 gene may further modulate these pathogenic effects by altering miR‐21 expression and activity. SNPs in miRNAs‐coding regions have been shown to disrupt miRNAs processing, target binding, and gene silencing efficiency, leading to changes in downstream biological processes [11, 12]. For instance, the rs1292037 SNP has been implicated in several diseases, such as coronary heart disease and type 2 diabetes, where it influences susceptibility and prognosis through its effects on miR‐21 expression [17, 18]. Although there is no direct evidence linking rs1292037 to T1DM, studies on other autoimmune and inflammatory diseases suggest that it may play a similar role by regulating key processes like β‐cell apoptosis and immune activation [14, 20].

Bioinformatics analyses have predicted numerous targets of miR‐21, including genes associated with apoptosis (e.g., PDCD4 and BCL2), inflammation (e.g., SMAD7 and TIMP3), and β‐cell function (e.g., PPP1R1A and SOX5) [9, 19]. Altered expression of these genes, driven by miR‐21 overexpression, may contribute to hallmark T1DM processes such as β‐cell death, inflammatory damage, impaired insulin secretion, and T‐cell‐mediated cytotoxicity [9, 19].

Given these findings, our study aims to explore the association between the rs1292037 SNP and T1DM susceptibility in the northern Iranian population. By investigating its impact on miR‐21 expression and its downstream targets, we seek to uncover the genetic and molecular mechanisms linking this SNP to T1DM pathogenesis. This approach not only enhances our understanding of miR‐21's role in T1DM but also underscores the importance of rs1292037 as a potential biomarker for early diagnosis and targeted therapeutic strategies.

## 2 Materials and Methods

### 2.1 Subjects

**Figure 1:**
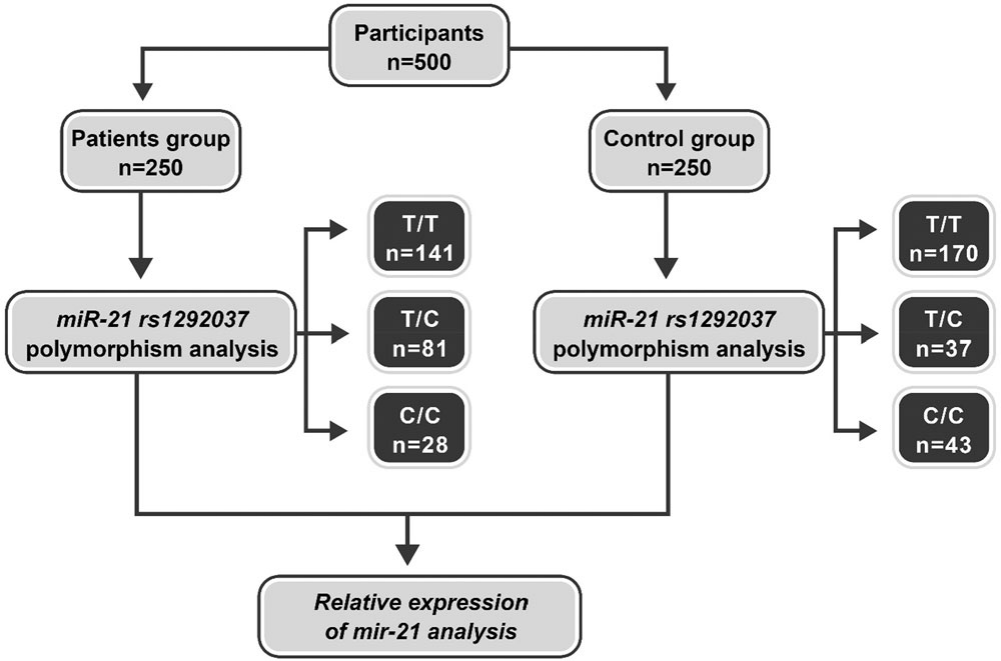
Flow diagram illustrating the conduction of the experimental study.

In this case‐control study, we assessed all patients with T1DM (case group) who were referred to the Endocrinology clinic at 17 Shahrivar Hospital (Rasht, Iran) from March 2019 to March 2020. Besides, the same number of healthy children as a control group were compared with cases. The groups were matched for age and sex. The flowchart of information on the participants in this study is shown in Figure 1. The study was performed according to the principles of the 1964 Helsinki Declaration and its further amendments and was approved by the ethical committee of Guilan University of Medical Sciences (Approval ID: IR.GUMS.REC.1397.427, Date: 2019‐02‐04). Parents/guardians of all subjects, including patients and controls, signed written informed consent. T1DM was diagnosed according to the latest criteria published by the American Diabetes Association [23]. Inclusion criteria for the case group were at least 6 months of T1DM and concomitant insulin injection. Patients with severe complications of T1DM such as nephropathy were excluded. Data were gathered through an interview by a checklist containing variables such as age, sex, body mass index (BMI), age of diagnosis, family history of diabetes, polyuria, polydipsia, weight loss, and abdominal pain. Peripheral blood samples (3 mL) were collected early in the morning from the participants at 17 Shahrivar Hospital in Rasht. To prevent coagulation, the blood samples were stored in EDTA‐containing tubes. Considering that RNA molecules, due to the presence of a free OH group at the 2'‐carbon of their sugar backbone, undergo rapid self‐catalyzed nucleophilic attack and degradation at room temperature, the blood samples were immediately transferred to the laboratory in a flask containing liquid nitrogen. RNA extraction was performed promptly. Additionally, DNA was extracted from the blood samples using the buffer‐detergent method to genotype the rs1292037 polymorphic site of the miR‐21 gene. The Hemoglobin A1C was extracted from their latest examination. BMI was calculated by dividing weight in kilograms to height in squared meter. Then, the mean values for both metrics were analyzed to assess the patient's overall health status.

### 2.2 Genotyping

Genomic deoxyribonucleic acid (DNA) was extracted from peripheral blood samples of all subjects using Triton X‐100. The concentration and purity of the extracted DNA were assessed using a NanoDrop spectrophotometer (Thermo Fisher Scientific, USA). Genotyping of rs1292037 was performed by polymerase chain reaction (PCR) followed by restriction fragment length polymorphism (RFLP). Forward and reverse primers of miR‐21 *rs1292037* were 5'‐ACTGTCTGCTTGTTTTGCCTA‐3' and 5'‐ TGAAAGAGATGAACCACGACT‐3', respectively. The PCR amplification was performed in a 20 µL reaction volume consisting of 3 µL of the extracted DNA (30 ng/µL), 10 µL of Taq DNA polymerase Master Mix Red (Ampliqon, Denmark), and 1 µL (10 pmol/µL) of each primer (forward and reverse). Samples were amplified in an MJ Mini thermal cycler (Bio‐Rad, USA). The following PCR conditions were used: an initial denaturation step at 94°C for 5 min, amplification for 35 cycles at 94°C for 45 s, 57°C for 45 s, and 72°C for 45 s, followed by an extension step at 72°C for 5 min. The PCR product (540 bp) was then analyzed by 1% agarose gel electrophoresis in Tris‐boric acid‐EDTA buffer and stained with RedSafe Nucleic Acid Staining Solution (Boca Scientific, USA). To detect allelic variation, amplicons were digested with TspRI restriction enzyme (Thermo Fisher Scientific, USA) according to the manufacturer's protocol. After digestion with TspRI, the PCR product was cleaved into 338‐bp and 202‐bp fragments in the presence of the C allele, while the T allele remained uncleaved (540‐bp). To investigate the association between the rs1292037 polymorphism in miR‐21 and T1DM, we analyzed the genotypes using several models. The codominant model examined each genotype (T/T, T/C, and C/C) independently to determine whether specific genotypes were associated with T1DM. The dominant model grouped the T/C and C/C genotypes together and compared them against the T/T genotype, allowing us to assess whether carrying at least one C allele influences T1DM risk. Conversely, the recessive model compared only the C/C genotype to the combined T/T and T/C genotypes to explore if two copies of the C allele are necessary to increase disease susceptibility. Additionally, we used an Overdominant model to specifically evaluate the T/C heterozygous genotype compared to both homozygous genotypes (T/T and C/C), thereby investigating whether the heterozygous form has a unique impact on disease risk. Furthermore, the allelic analysis compared the overall frequency of T and C alleles between patients and controls, providing broader insights into the role of each allele in T1DM susceptibility. Finally, the enzyme‐digested products were then separated on a 2% agarose gel and stained with RedSafe.

### 2.3 Real‐Time PCR

Ribonucleic acid (RNA) was isolated from the peripheral blood using the SanPrep Column microRNA Mini‐Prep Kit (Bio Basic, Canada) according to the manufacturer's instructions and quantified using a UV‐spectrophotometer (Thermo Fisher Scientific, USA). In addition, RNA integrity was analyzed using 2% agarose gel electrophoresis. Complementary DNA (cDNA) was synthesized using a RevertAid™ First Strand cDNA Synthesis Kit (Thermo Fisher Scientific, USA) with a miR‐21 stem‐loop primer (5'‐ GTCGTATCCAGTGCAGGGTCCGAGGTATTCGCACTGGATCAACA‐3'), according to the manufacturer's instructions. This was followed by reverse transcription‐ PCR (RT‐PCR).

Quantitative real‐time PCR (qRT‐PCR) was performed to evaluate the expression level of miR‐21 in T1DM patients and controls. Genotyping was performed in a 20 µL reaction mixture consisting of 1 µL cDNA (2 µg/µL), 10 µL SYBR‐Green 2X Mastermix (TB Green Premix Ex Taq II Tli RNase H Plus; Takara, Japan), 1 µL (5 pmol/µL) of each specific forward and reverse primer. Amplification was performed using an MJ Mini Thermal Cycler (Bio‐Rad, USA). Cycling conditions were 5 min of incubation at 95°C, 45 cycles at 95°C for 30 s, 63°C for 30 s, and 72°C for 30 s, followed by a 5‐min extension at 72°C. The U48 was used as an endogenous control to normalize the expression level of miR‐21. The used primers are as follows: *miR‐21* (F): 5'‐GGTGTAGCTTATCAGACTGATG‐3', *miR‐21* (R): 5'‐AGGGTCCGAGGTATTCGC‐3', *U48* (F): 5'‐ GAGTGATGATGACCCCAGGTAA‐3', and *U48* (R): 5'‐ GTGCAGGGTCCGAGGT‐3'. All reactions were carried out in triplicate. Fold changes in the expression levels were calculated using the 2^−ΔΔCT^ method [24]. All assays were blinded and performed by two researchers who were unaware of the case or control status. Five percent of the samples were randomly repeated for quality control.

### 2.4 Statistical Analysis

The chi‐squared test (*χ* ^2^) was used to assess differences in genotype and allele distribution between T1DM patients and healthy controls. Hardy‐Weinberg equilibrium (HWE) was assessed by a goodness‐of‐fit *χ* ^2^ test. Odds ratios (ORs) with 95% confidence intervals (CIs) were used to assess the strength of the association. *P*‐values less than 0.05 were considered statistically significant. Data related to SNP genotyping were analyzed using MedCalc statistical software (version 19.5.3; Belgium). All other data and information were analyzed and examined using GraphPad Prism (version 8.0.2, USA). Significance levels were established a priori, and statistical tests were conducted as two‐sided.

### 2.5 Bioinformatics Analysis

The potential targets of miR‐21 were investigated using five different bioinformatics web servers: (1) PicTar which uses genome‐wide alignments according to the genome of eight vertebrates [25], (2) miRTarBase which is a database that predicts the miRNA targets based on the experimentally validated miRNA‐mRNA interactions [26], (3) TargetScanHuman, which uses experimental datasets as well as canonical binding sites for miR‐21 targets [27], (4) miRDB which uses sequence alignments and text mining for efficient target prediction [28], and (5) MirSNP which calculates the binding energy of mRNA‐miRNA interactions at canonical binding sites (and the effect of their corresponding SNPs) [29]. For PicTar, a score of 1.00 was chosen as the cut‐off for interactions. For miRTarBase, predictions validated by at least three methods were selected. For TargetScanHuman, targets with a cumulative weighted context++ score of −0.50 or more negative were selected. For miRDB, a target score of 80 or higher was used. Finally, for MirSNP, only the targets with a binding energy of ‐23.00 or lower were investigated. Targets predicted by more than two databases were selected for further investigation.

## 3 Results

### 3.1 Features of the Study Subjects

The study included a total of 500 participants, divided into 250 patients with T1DM and 250 control subjects, matched for age and sex (Table 1). There was no significant difference in age between the T1DM group and controls, with a mean age of 10.26 ± 3.57 years for T1DM patients and 10.81 ± 3.58 years for controls (*p* = 0.11). The groups were also balanced in terms of sex distribution, with 45.6% of T1DM patients and 46.4% of controls being male, which was not statistically significant (*p* = 0.19).

**Table 1:** Clinical and biochemical characteristics in cases and controls.

Variables	Cases n (%)	Controls n (%)	*p*‐value
Age	10.26 ± 3.57	10.81 ± 3.58	0.11
*Sex*			
Male	114 (45.6%)	116 (46.4%)	0.19
Female	136 (54.4%)	134 (53.6%)	0.07
BMI^a^	16.67 ± 1.61	17.00 ± 1.60	0.29
Hemoglobin A1C	9.64 ± 1.65	5.13 ± 0.26	< 0.0001
Age of Diagnosis	8.119 ± 3.69		
*Family History of Diabetes*			
Type 1	24 (9.6%)	3 (1.2%)	< 0.0001
Type 2	137 (54.8%)	15 (6%)	< 0.0001
None	89 (35.6%)	232 (92.8%)	< 0.0001
*Polyuria*			
Yes	250 (100%)		
No	0 (0%)		
*Polydipsia*			
Yes	242 (96.8%)		
No	8 (3.2%)		
*Weight Loss*			
Yes	234 (93.6%)		
No	16 (6.4%)		
*Abdominal Pain*			
Yes	161 (64.4%)		
No	89 (35.6%)		
*Lethargy*			
Yes	153 (61.2%)		
No	97 (38.8%)		

^a^ BMI= Body mass unit

In BMI, there was no significant difference between the two groups; the mean BMI was 16.67 ± 1.61 for T1DM patients and 17.00 ± 1.60 for controls (*p* = 0.29). However, a significant difference was observed in hemoglobin A1c (HbA1c) levels, a key indicator of glycemic control. T1DM patients exhibited a markedly higher HbA1c level of 9.64 ± 1.65 compared to 5.13 ± 0.26 in the control group (*p* < 0.0001), reflecting poor glycemic control in the patient group.

The average age of diagnosis in T1DM patients was 8.12 ± 3.69 years, indicating early onset of the disease. Additionally, a family history of diabetes showed significant variations between the two groups. A family history of type 1 diabetes was reported in 9.6% of T1DM patients, compared to only 1.2% in controls (*p* < 0.0001). Similarly, a family history of type 2 diabetes was significantly more common in T1DM patients (54.8%) than in controls (6%) (*p* < 0.0001). Only 35.6% of T1DM patients reported no family history of diabetes, compared to 92.8% of controls (*p* < 0.0001).

Clinical symptoms associated with T1DM, such as polyuria, polydipsia, and lethargy, were prevalent in the patient group. All T1DM patients (100%) experienced polyuria, while polydipsia was present in 96.8% of patients. Additionally, 93.6% of T1DM patients reported significant weight loss, and 64.4% experienced abdominal pain. Lethargy was also common, affecting 61.2% of T1DM patients. These symptoms were not reported in the control group, underscoring the marked clinical differences between the groups.

### 3.2 rs1292037 and T1DM

The distribution of miR‐21 rs1292037 genotypes and alleles among T1DM patients and control subjects is summarized in Table 2. Analysis under a codominant model revealed a significant association of the T/C genotype with T1DM, observed in 32.4% of patients versus 14.8% of controls (OR = 2.63, 95% CI = 1.68–4.13, *p* < 0.0001). The C/C genotype, however, showed no significant association (OR = 0.78, *p* = 0.36 *p* = 0.36). In a dominant model, T/C + C/C genotypes were more common in T1DM patients than controls (43.6% vs. 32%; OR = 1.64, 95% CI = 1.14–2.36, *p* = 0.007). Conversely, under a recessive model, no significant association was observed for the C/C genotype (OR = 0.6, 95% CI = 0.36–1.01, *p* = 0.056). Further analysis in an overdominant model indicated a substantial increase in the risk of T1DM for individuals with the T/C genotype (OR = 2.74, 95% CI = 1.78–4.27, *p* < 0.0001). Additionally, the C allele itself was significantly more frequent among patients compared to controls (27.4% vs. 24.6%; OR = 1.36, 95% CI = 1.03–1.8, *p* = 0.02).

**Table 2:** Genetics of miR‐21 SNP in patients and controls.

Model	Patients N (%)	Controls N (%)	OR (95% CI)
Codominant genotype			
T/T	141 (56.4%)	170 (68%)	1.00
T/C	81 (32.4%)	37 (14.8%)	2.63 (1.68–4.13)^a^
C/C	28 (11.2%)	43 (17.2%)	0.78 (0.46–1.32)^b^
Dominant genotype			
T/T	141 (56.4%)	170 (68%)	1.00
T/C+C/C	109 (43.6%)	80 (32%)	1.64 (1.14–2.36)^c^
Recessive genotype			
T/T+T/C	222 (88.8%)	207 (82.8%)	1.00
C/C	28 (11.2%)	43 (17.2%)	0.6 (0.36–1.01)^d^
Overdominant genotype			
T/T+C/C	169 (67.6%)	213 (85.2%)	1.00
T/C	81 (32.4%)	37 (14.8%)	2.74 (1.78–4.27)^a^
Allele			
T	363 (72.6%)	377 (75.4%)	1.00
C	137 (27.4%)	123 (24.6%)	1.36 (1.03–1.8)^e^

^a^ *p* < 0.0001, ^b^ *p* = 0.36, ^c^ *p* = 0.007, ^d^ *p* = 0.056, ^e^ *p* = 0.02. CI = confidence interval, OR = odds ratio.

### 3.3 miR‐21 Expression in T1DM

**Figure 2:**
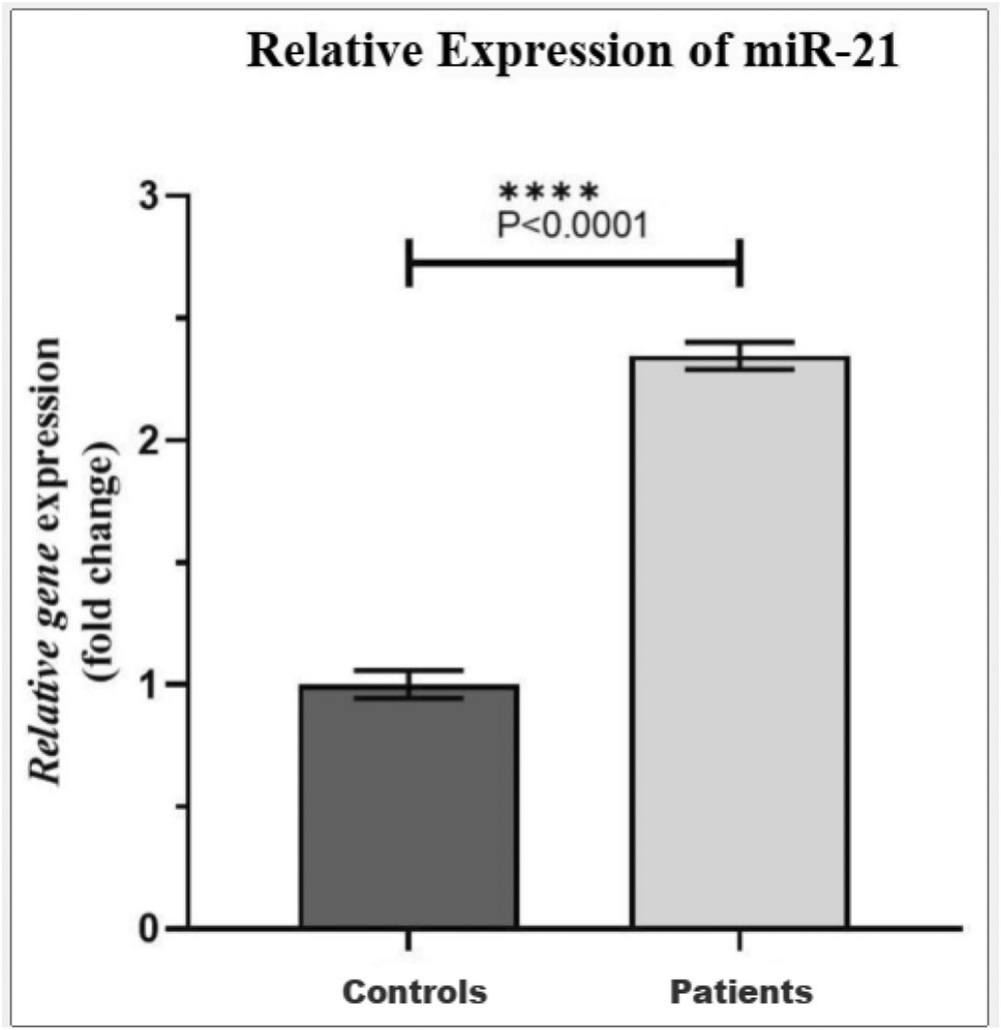
Relative gene expression of miR‐21 in control subjects and T1DM patients.

**Figure 3:**
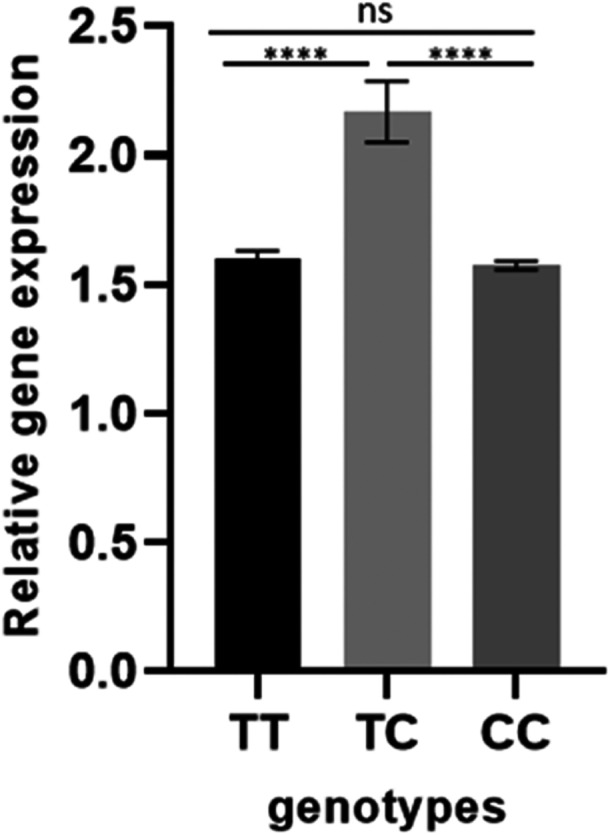
Relative gene expression of miR‐21 in subjects carrying the T/T, T/C, or C/C genotypes.

The qRT‐PCR was used to determine the relative expression of the miR‐21 gene in the T1DM and control groups. The data obtained showed a significant difference in the expression level of miR‐21 between T1DM and normal controls. As shown in Figure 2, the mean expression level of miR‐21 in the control subjects and T1DM patients was calculated to be 1 and 2.34, respectively. The results indicated that the miR‐21 gene is significantly upregulated in T1DM patients by almost twofold compared to the control group (*p* < 0.0001). As Figure 3 shows, miR‐21 expression was significantly upregulated when carrying the T/C genotype. Hence, those carrying this genotype had significantly higher expression compared to those with T/T (*p* < 0.0001) or C/C (*p* < 0.0001).

### 3.4 Functional Roles of miR‐21 in T1DM: Bioinformatics Analysis

Based on our evaluation of five servers, we found that miR‐21 targets 184 genes. Therefore, we selected these genes for in‐depth evaluation (see Supporting Information S1: Table T1). Of these targets, 64 genes were predicted by two or more than two databases. Furthermore, a review of the existing literature revealed that 14 of these genes are commonly downregulated in diabetes with identified clinical consequences (Table 3).

**Table 3:** miR‐21 target genes and functional consequences of their downregulation in T1DM.

Target	Databases	Consequences in diabetes
**BCL2**	miRTarBase, miRDB	Induces the mitochondrial pathway of apoptosis in β‐cells
**CDC25A**	PicTar, miRTarBase	Impaired cell cycle progression and mesangial cell hypertrophy
**CPEB2**	miRTarBase, miRDB	Induces inflammatory response in Diabetic patients
**E2F3**	PicTar, miRTarBase	Induces podocyte injury which can lead to Diabetic Nephropathy
**FASLG**	miRTarBase, TargetScanHuman, miRDB	Inhibition of the extrinsic pathways of apoptosis in diabetogenic cytotoxic T cells
**NFIB**	PicTar, miRTarBase	Downregulation of FOXA1 and podocyte injury
**PDCD4**	PicTar, miRTarBase, miRDB	β‐cell apoptosis
**PLAG1**	PicTar, miRDB	β‐cell apoptosis
**PPARA**	PicTar, miRTarBase	Downregulation of NRF2 and increased sensitivity of β‐cells to oxidative stress
**PPP1R1A**	TargetScanHuman, miRDB	Reduced insulin production in β‐cells
**SMAD7**	PicTar, miRDB	Induction of renal fibrosis and renal inflammation in diabetic patients
**SOX5**	PicTar, miRTarBase	Reduced insulin production and secretion
**TIMP3**	PicTar, miRTarBase	Inflammatory response through upregulation of STAT pathway [fiorentino]
**ZDHHC17**	PicTar, MirSNP	β‐cell apoptosis and inflammation

Among these candidate targets, downregulation of BCL2, PDCD4, PLAG1, PPARA, and ZDHHC17 is associated with beta cell death [30, 31, 32, 33, 34]. The downregulation of CDC25A, E2F3, and NFIB increases the risk of developing diabetic nephropathy [35, 36, 37]. Reduced expression of CPEB2, SMAD7, and TIMP3 leads to inflammatory responses in diabetic patients [38, 39, 40]. Downregulation of PPP1R1A and SOX5 is associated with reduced insulin production or secretion [41, 42]. Finally, suppression of FASLG can lead to cytotoxic behavior of T cells (Table 3) [43].

## 4 Discussion

T1DM is one of the most common chronic diseases in children, which is mainly characterized by insulin deficiency [44]. The miRNAs, as regulators of gene expression, are thought to contribute to several pathological conditions including T1DM [45]. SNPs in the miRNA‐coding gene have been reported to affect the biological function of these noncoding RNAs, which may lead to the onset of many human diseases [46]. A number of studies have investigated the role of miRNA‐related SNPs in the incidence and prognosis of T1DM. For example, miR‐196a rs11614913 has been reported to be associated with the pathogenesis of T1DM [47]. In the present study, 250 T1DM patients and 250 population‐matched controls were selected for the evaluation of *miR‐21* (*rs1292037*) SNP frequency and miR‐21 expression. Overall, the C allele of the polymorphic site was found to be more common in T1DM patients.

Although miR‐21 *rs1292037* has been shown to correlate with the prognosis in hepatocellular carcinoma and cervical cancer [14, 48], there is no reported evidence of an association between this reference SNP and T1DM. It is observed that miR‐21 targets genes involved in cell proliferation and cell apoptosis, including PDCD4 [31]. In addition, to the critical role of miR‐21 SNPs in the incidence and prognosis of autoimmune diseases such as T1DM, alteration in the expression level of miRNAs in autoimmune diseases has been manifested by several researchers. Osipova et al. have shown that the circulating level of miR‐21 is significantly upregulated in the urine and plasma of pediatric patients with T1DM, which is consistent with our findings [49]. Mostahfezian et al. have also reported that miR‐21 is upregulated in T1DM patients [20]. It has been postulated that upregulated levels of miR‐21 inhibit T‐cell apoptosis, leading to autoimmunity. Compelling evidence has shown that upregulation of miR‐21 can expose β‐cells to pro‐inflammatory cytokines. This unfortunate phenomenon promotes cell death, which stimulates T1DM [50]. Our study showed that miR‐21 is significantly overexpressed in T1DM patients compared to controls. Additionally, the T/C genotype contributes to the highest expression rate of miR‐21 compared to the T/T and C/C genotypes among the cases studied, suggesting a genotype‐dependent effect on miR‐21 levels. This overexpression may exacerbate T1DM pathogenesis through several mechanisms: it promotes the survival of autoreactive T‐cells by inhibiting apoptosis‐related genes like PDCD4, enhances inflammatory cytokine production via the STAT3 and NF‐κB pathways, and increases beta‐cell susceptibility to cytokine‐induced apoptosis. The T/C genotype's association with higher miR‐21 expression implies that individuals with this genotype may experience more intense inflammatory and apoptotic responses, accelerating the autoimmune destruction of pancreatic beta cells. These findings underscore the biological significance of the T/C genotype and miR‐21 overexpression as potential contributors to T1DM onset and progression.

Bioinformatic analysis of miR‐21 target prediction reveals its extensive impact on multiple genes involved in pathways critical to the development and progression of T1DM, suggesting that overexpression of this miRNA may be associated with β‐cell death, diabetic nephropathy, inflammatory responses, impaired insulin production or secretion, and T‐cell cytotoxicity. As shown in Table 3 and Supporting Information S1: Table T1, several predicted target genes are implicated in hallmark processes of T1DM, including β‐cell apoptosis, inflammation, and impaired insulin production. For instance, PDCD4 and BCL2, two key antiapoptotic genes, are predicted to be downregulated by miR‐21 overexpression. Their suppression could exacerbate pancreatic β‐cell apoptosis via intrinsic and extrinsic apoptotic pathways, aligning with the established role of β‐cell death in T1DM onset [30, 31]. Additionally, ZDHHC17 and PLAG1, also targeted by miR‐21, further contribute to β‐cell vulnerability [32, 34].

Genes regulating inflammation, such as SMAD7 and TIMP3, are also predicted targets of miR‐21. The downregulation of SMAD7 can enhance renal fibrosis and inflammation, potentially increasing susceptibility to diabetic nephropathy, a major complication of T1DM. Similarly, reduced TIMP3 expression has been shown to enhance pro‐inflammatory cytokine production via the STAT and NF‐κB pathways, intensifying β‐cell destruction [39, 40]. These findings underscore miR‐21's potential role in exacerbating inflammatory responses and contributing to complications such as nephropathy in T1DM [51, 52].

The upregulation of miR‐21 observed in T1DM patients, particularly those carrying the T/C genotype (Figure 3), highlights its potential as a biomarker for disease progression and severity. Elevated miR‐21 levels could indicate heightened inflammatory responses, β‐cell apoptosis, or insulin production impairment, suggesting its utility in early detection or disease stratification. Furthermore, key targets of miR‐21, such as PPP1R1A and SOX5, directly affect insulin secretion and β‐cell function. Therapeutic strategies aimed at restoring their expression may help mitigate β‐cell dysfunction and maintain glucose homeostasis [41, 44]. Early detection of T1DM not only improves patients' quality of life but also increases the possibility of preventing complications such as heart disease, foot ulcers, retinopathy, and neuropathy [53]. More importantly, early detection can positively impact the honeymoon period, a transitional remission phase where insulin doses can be significantly reduced or even stopped altogether [54].

Additionally, FASLG, a regulator of T‐cell cytotoxicity, is suppressed by miR‐21 overexpression. This suppression may impair apoptosis in autoreactive T cells, exacerbating autoimmune destruction. These findings position miR‐21 and its targets as promising candidates for therapeutic intervention, either by targeting miR‐21 itself or modulating its downstream effects [43]. Future studies should focus on validating these bioinformatics predictions experimentally to clarify the precise roles of miR‐21 targets in T1DM. Longitudinal analyses could assess miR‐21 expression at different disease stages to determine its utility as a prognostic biomarker.

From a therapeutic standpoint, targeting miR‐21 with antagomiRs or other inhibitors could restore the expression of protective genes, such as PDCD4 and BCL2, potentially delaying disease progression. Investigations into the role of miR‐21 in modulating immune pathways, particularly through the NF‐κB and STAT3 axes, could further elucidate its contribution to the autoimmune and inflammatory processes driving T1DM [30, 31, 40]. The analysis of miRNA expression levels and miRNA‐related SNPs has also been proposed as a biomarker in medicine and biotechnology for the early detection of diseases with genetic backgrounds [51, 52].

In summary, the interplay between miR‐21 and its predicted targets underscores its dual role as a biomarker and therapeutic target in T1DM. The findings of this study not only enhance our understanding of T1DM pathogenesis but also pave the way for new diagnostic and treatment strategies.

Despite the significant findings of this study, several limitations must be acknowledged. Firstly, the sample size, though substantial, may still not be sufficient to generalize the results to broader populations. A larger cohort across multiple regions and ethnic groups would provide more robust and universally applicable conclusions. Secondly, this study is geographically restricted to an Iranian population, which may limit the generalizability of the findings to other ethnicities and environmental contexts where genetic and epigenetic factors influencing T1DM might differ. Thirdly, while the study provides insights into the association between miR‐21 rs1292037 polymorphism and T1DM, it does not establish causality. Further studies are needed to explore the exact mechanisms underlying these associations. Lastly, the bioinformatics analysis, though comprehensive, was reliant on predictions and literature data; experimental validation of miR‐21 target genes and their roles in T1DM pathogenesis is necessary for confirming these findings.

In conclusion, after genotyping 250 patients with T1DM and 250 controls, we found that the miR‐21 rs1292037 T/C genotype is significantly associated with the incidence of T1DM. In addition, miR‐21 is significantly overexpressed in T1DM patients compared to controls. Regarding miR‐21 predicted target genes, overexpression of miR‐21 may be associated with beta cell death, diabetic nephropathy, inflammatory responses, impaired insulin production or secretion, and T‐cell cytotoxicity, all of which are important in the initiation and progression of T1DM.

## Author Contributions

**Reza Bayat:** investigation, data curation, resources, formal analysis, writing – original draft, writing – review and editing. **Zivar Salehi:** conceptualization, supervision, methodology, resources, formal analysis writing – original draft, writing – review and editing. **Setila Dalili:** conceptualization, supervision, data acquisition, resources, writing – original draft, writing – review and editing. **Farhad Mashayekhi:** methodology, writing – original draft, writing – review and editing. All authors have read and approved the final version of the manuscript. Zivar Salehi had full access to all of the data in this study and takes complete responsibility for the integrity of the data and the accuracy of the data analysis.

## Ethics Statement

The study was performed according to the principles of the 1964 Helsinki Declaration and its further amendments and was approved by the ethical committee of Guilan University of Medical Sciences (Approval ID: IR. GUMS. REC.1397.427, Date: 2019‐02‐04).

## Consent

Parents/guardians of all subjects, including patients and controls, signed written informed consent.

## Conflicts of Interest

The authors declare no conflicts of interest.

### 1 Transparency Statement

The lead author Zivar Salehi affirms that this manuscript is an honest, accurate, and transparent account of the study being reported; that no important aspects of the study have been omitted; and that any discrepancies from the study as planned (and, if relevant, registered) have been explained.

## Supporting information

Supporting information.

## Data Availability

The data that support the findings of this study are available from the corresponding author upon reasonable request. Our data cannot be shared openly to protect study participants' privacy and our country's rules and regulations on genetic data, but we have Real‐time PCR gene expression output data and the image of agarose gel for polymorphism. Therefore, in case of any formal request, we can only send some of them as related files for review and we cannot submit them as Supporting files to be available after publication.
