# Supplementary material for: Influence of rs1292037 Genetic Variant on miR‐21 Gene Expression in Patients With Type 1 Diabetes Mellitus: A Case‐Control Study
Source: Health Sci Rep. 2025 Mar 2;8(3):e70480. doi: 10.1002/hsr2.70480 (PMC11872810; doi:10.1002/hsr2.70480)
Supplement: Supplementary file 1 — Supporting information. [file HSR2-8-e70480-s001.docx]

**T1. miR-21 targets according to PicTar, miRTarBase, TargetScanHuman, miRDB, and MirSNP.**

| **Server** | **Total** | **Gene Symbol** |
| --- | --- | --- |
| PicTar | 77 | TAGAP RAB6C H63 SOX7 PITX2 MAMDC1 BTBD3 MATN2 TRPM7 MATR3 PURB PCBP1 KIAA2024 EHD1 BRD2 SMARCD1 RASA1 ATXN10 ARHGEF7 STAG2 CHD7 HBP1 TAF5 DLX2 NBEA ACVR2 ASF1A PACS1 CRIM1 ADNP SLC9A6 FBXO11 M-RIP TESK2 TRIM2 ACBD5 ARHGAP24 TRIM9 KBTBD6 SFRS8 SPIN N-PAC BNC2 SET8 RP2 MAPRE1 CNTFR LEMD3 MRPL9 PB1 NTF3 ARMC8 SPG20 LOC51136 EPHA4 WWP1 HIP2 FBXL17 RNF111 SATB1 PCSK6 SOX2 BRD1 RNF103 GRIA2 BAHD1 MGC4796 SSFA2 KIAA1468 MAK3 GANC CREBL2 DDA3 HNRPK ABCD2 ASPN PDZK3 |
| miRTarBase | 80 | DIMTL1 MCM3 LAMB3 SSRP1 PTEN CDK6 MNT TGFBR2 SERPINB5 PTPN1 TGFBR3 ANKRD46 FGFR1 RPS7 GJA1 RAD52 BMPR2 MECP2 ISCU ROBO1 XIAP APAF1 AP1S2 EFNA3 SDHD BIRC5 SERPINI1 NFIB EHD2 PEA15 EGFR SIRT1 IKBKB TPM1 VOPP1 P4HB IGF2R EZH2 MSH6 IL11 HDGF NFAT5 DDIT3 BDNF IGFBP3 DDAH1 DERL1 BECN1 GALNT7 MAT2B ACTN1 XIST PDE3A ELOVL6 WNK1 MAPK8 PLXNB1 RICTOR CPEB4 MTAP E2F1 MUC4 DAXX NFE2L1 NCAM1 TCF12 LASP1 POU2F2 GPD1L INPP5A M6PR MAT2A ALDH5A1 IGFBP5 RB1 TOB1 MSH2 TOPORS PIM1 FGFRL1 |
| TargetScanHuman | 19 | MYBPH OFCC1 HTN1 RINT1 HIGD1A OARD1 RSRC2 SHISA7 RBM33 CCAR1 SNRBP2 TFRC PHYHD1 AIM1L GPR64 TFG BRWD1 OR5T2 RPA3-AS1 |
| miRDB | 51 | SRSF2 VCL PRDM11 STK38L MALT1 CREBRF CDK14 ZIC5 KDM7A RC3H1 LAMP1 NKIRAS1 TMEFF1 FSBP GID4 BCL7A GAD2 RBPJ ATP1B1 CDK8 TSC22D2 OSR1 UMAD1 HS2ST1 SKP2 PHYHIPL ADGRG2 HSD17B4 PARD3B ZBTB41 SCML2 GATAD2B AKAP11 CAMSAP2 GPATCH2L TMEM164 MAP3K1 DDX4 RALGPS2 SNRK SEH1L GPM6A TOGARAM1 USP51 PDZD2 UBE2D RIPOR3 FAM13A PBRM1 MBNL3 FAM83H |
| MirSNP | 39 | DCX HAUS5 ATAT1 ACSS1 CYCS POLR3A MLLT1 PHLPP2 BRCA1 CLN8 LIMS2 NKX2-8 SHROOM2 TBC1D20 CUX1 BMI1 STX7 NAA38 CHST15 CABLES2 MBOAT1 OPCML PHC3 DSG1 CXCL12 PRUNE2 DCLK1 AMACR SNX20 GBP6 SHB KLHL3 BEND4 COMMD3 CISD3 EVL APEB1 CHMP1A SMOC2 |
| PicTar TargetScanHuman miRTarBase | 1 | ARID1A |
| PicTar  miRDB  miRTarBase | 3 | STAT3 PDCD4 RECK |
| PicTar TargetScanHuman miRDB | 4 | YOD1 PLEKHA1 PELI1 CCL1 |
| TargetScanHuman miRDB  miRTarBase | 3 | FASLG FGF18 IL12A |
| PicTar miRTarBase | 13 | BTG2 PPARA RAB11A TIMP3 SOX5 RASGRP1 NDUFA4 EIF1AX RHOB E2F3 JAG1 SPRY2 CDC25A |
| PicTar TargetScanHuman | 1 | CCM1 |
| PicTar miRDB | 12 | SMAD7 SPRY1 YAP1 RAB6A PLAG1 CPEB3 SKI TGFBI ELF2 CASKIN1 PAN3 PCDH17 |
| MirSNP PicTar | 8 | TNFSF6 SLC7A6 IGSF4D PPP3CA KCNA3 PIP3AP KIAA1194 ZDHHC17 |
| TargetScanHuman miRTarBase | 1 | ARL2 |
| miRDB miRTarBase | 8 | RTN4 SERINC3 FOXN3 CPEB2 BCL2 ALCAM MAP2K3 TIAM1 |
| MirSNP miRTarBase | 2 | SH3BGRL TP63 |
| TargetScanHuman miRDB | 9 | FYTTD1 RSAD2 KRIT1 PPP1R1A MAGEB18 NAP1L5 OR10W1 SLC35B3 RRAGB |
| MirSNP TargetScanHuman | 1 | DEFA4 |
| MirSNP miRDB | 1 | KLF3 |
